# Pancreatic adenocarcinoma in a patient with Situs Inversus: a case report of this rare coincidence

**DOI:** 10.1186/1477-7819-7-98

**Published:** 2009-12-18

**Authors:** Eric L Sceusi, Curtis J Wray

**Affiliations:** 1 Department of Surgery, University of Texas Medical School at Houston, Houston, Texas, USA

## Abstract

**Background:**

*Situs inversus *(SI) is a relatively rare occurrence in patients with pancreatic adenocarcinoma. Pancreatic resection in these patients has rarely been described. CT scan imaging is a principle modality for detecting pancreatic cancer and its use in SI patients is seldom reported.

**Case Presentation:**

We report a 48 year old woman with SI who, despite normal CT scan 8 months earlier, presented with obstructive jaundice and a pancreatic head mass requiring a pancreaticoduodenectomy. The surgical pathology report demonstrated pancreatic adenocarcinoma.

**Conclusion:**

SI is a rare condition with concurrent pancreatic cancer being even rarer. Despite the rarity, pancreaticoduodenectomy in these patients for resectable lesions is safe as long as special consideration to the anatomy is taken. Additionally, radiographic imaging has significantly improved detection of early pancreatic cancer; however, there continues to be a need for improved detection of small neoplasms.

## Background

*Situs inversus *(SI) occurs as the result of congenital chromosomal aberrations and results in reversal of the *right to left *orientation of the internal organs. The incidence of this phenomenon is approximately 1 in 10,000 [1]. Pancreatic adenocarcinoma is an aggressive malignancy and is the 4^th ^most common cause of cancer-related deaths in the USA [2]. Previous authors have described pancreaticoduodenectomy procedures in patients with SI. Macafee noted 30 case reports of cancers in SI patients since 1966 including four cases of pancreatic adenocarcinoma, three cholangiocarcinomas and two periampullary cancers prior to 2006 [3]. They also report that there is no data to suggest that SI patients are at increased risk of malignancy. Since that time there have been no new reported cases of pancreaticoduodenectomy in SI patients. We present a case of a patient with SI who developed a pancreatic mass and biliary obstruction requiring a pancreaticoduodenectomy despite having a normal CT scan 8 months prior to the development of symptoms.

A pancreas protocol CT scan of the abdomen is considered the single best study to evaluate for pancreatic neoplasms; however, it has limited ability to detect small lesions [4]. Early detection is the key to offering a potentially curative resection yet radiologic signs are often subtle and there are few reports describing the time interval between CT scan evidence and the development of pancreatic cancer. Our patient had a CT scan 8 months prior to the diagnosis of pancreatic cancer which showed some mild pancreatic atrophy, however she did not have evidence of a mass at that time.

## Case Presentation

**Figure 1:**
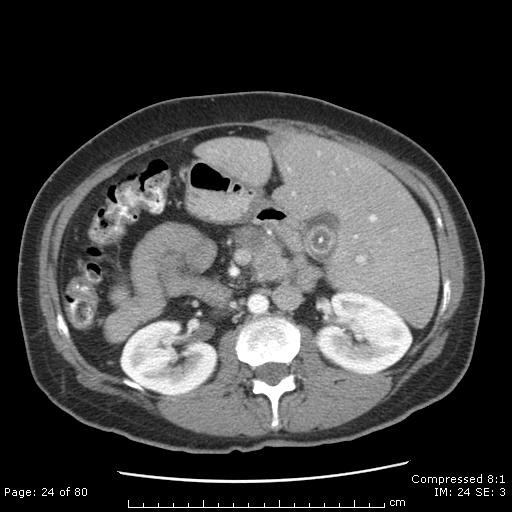
**Diagnostic CT scan**. CT scan obtained 8 months later when the patient presented with jaundice and a bulging Ampulla of Vater on ERCP. A new 4.2 cm mass is now present in the pancreatic head, obstructing the common bile duct.

**Figure 2:**
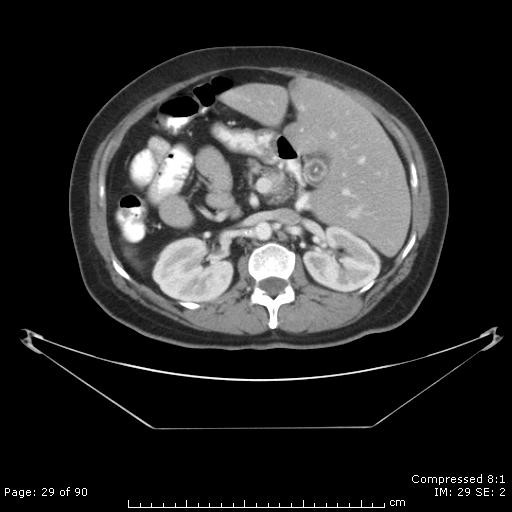
**Pre-diagnosis CT scan**. Consecutive CT scan slices demonstrate SI and mild atrophy of the pancreatic head but no mass present 8 months prior to her diagnosis of pancreatic cancer. The CT scan was obtained to evaluate abdominal pain.

A 48 year old Hispanic female presented to the emergency room with vague abdominal pain and new onset jaundice. Her stated past medical history was significant for diabetes mellitus, hypertension and asthma. Upon physical examination she was alert, afebrile and displayed significant jaundice. Abdominal examination revealed epigastric pain and a left upper quadrant mass. An ultrasound of the abdomen was performed to evaluate for gallbladder pathology, cholelithiasis and/or biliary tract dilation. Upon sonographic evaluation, it was noted that her intra-abdominal organs were not located in the normal anatomic position, including her liver in the left upper quadrant. A chest radiograph also revealed dextrocardia and the diagnosis of *situs inversus *(SI) was confirmed. Mild gallbladder wall thickening was noted as well as significant extrahepatic biliary duct dilation. Due to the level of jaundice and presumed biliary obstruction an ERCP was attempted, but was unsuccessful due to difficulty with cannulation of the inverted ampulla of Vater. The ampulla was also noted to be significantly protruding into the lumen of the duodenum, thus prompting a CT scan of abdomen. A 4.2 cm pancreatic head mass was discovered (Figure 1). Upon review of the electronic medical record, the patient had a previous CT scan of the abdomen 8 months earlier for vague abdominal pain which showed mild atrophy but no evidence of a pancreatic mass (Figure 2). Her serum CA19-9 (586 U/mL) was also elevated raising the suspicion for pancreatic cancer.

**Figure 3:**
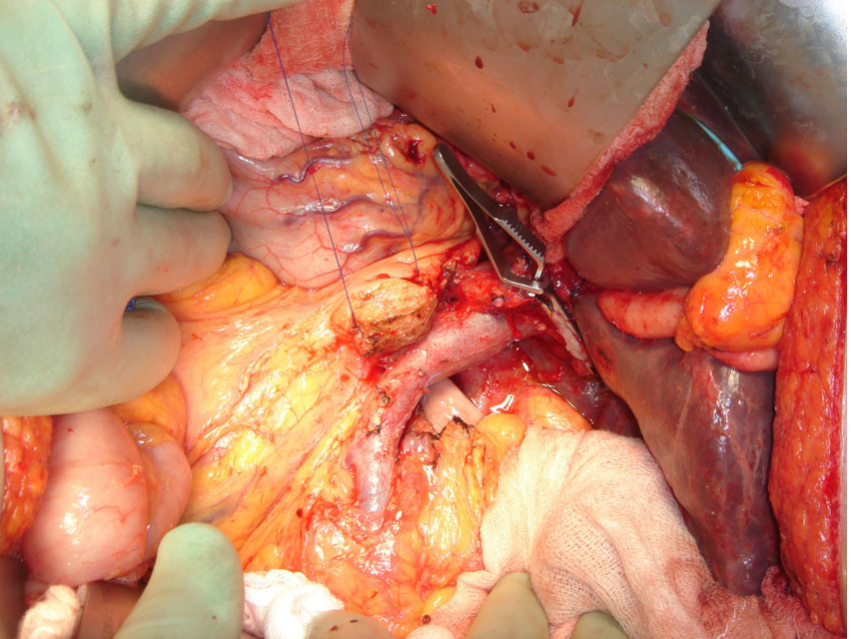
**Intra-operative photograph prior to reconstruction**. Abdominal contents noted to be inverted. Liver positioned in the right upper quadrant. Prolene stay sutures mark the cut edge of the pancreas. Bulldog clamp is occluding the common hepatic duct. Superior mesenteric vein and portal vein oriented to the patient's left.

Interventional radiology was able to perform a percutaneous transhepatic cholangiogram and place an external biliary catheter to decompress her biliary system. The patient subsequently underwent a pancreaticoduodenotomy and pathology showed moderately differentiated pancreatic ductal adenocarcinoma T3, N0, Mx (American Joint Committee on Cancer Stage IIa) (Figure 3). Follow-up CT scan 5 months post-operatively showed no evidence of recurrent cancer.

## Conclusions

Patients with SI generally present with a mirror image of their abdominal anatomy. During embryogenesis, the normal asymmetry of adult anatomy develops through three main pathways described by Kosaki and Casey [5]. The first utilizes lateralization of initially midline structures beginning with the rightward movement of the heart tube at developmental day 23, followed by the abdomen with stomach rotation beginning at 35 days and the rotation of the small and large intesting completing by day 77. Secondly, there is asymmetric regression and remodelling of embryonic veins. The third mechanism involves the continuation of early developmental asymmetry as exhibited by the development of the bronchial tree. About 20-25% of SI cases are associated ciliary dyskinesia syndromes and respiratory symptoms as part of the complex known as Kartagener syndrome [6], however, the cause of SI is currently unknown [7]. Mutations in genes responsible for lateralization and polarizations as well as alterations in the TGF-B family gene, Nodal and in the transcription factor HNF-3B are possibly involved in the process [5].

The safety of performing pancreaticoduodenectomy in SI patients has been established in prior reports by Macafee and Bilimoria. Special care must be exercised to identify the presence of several associated anatomic abnormalities, such as a midline gallbladder or liver, rotational abnormalities of the small and large intestine, interruption of the inferior vena cava, truncation of the pancreas or ipsilateral location of the aorta and IVC [8]. These findings are more common in patients who present with polysplenia and SI and can be identified with a thorough review of CT scans preoperatively and careful intra-operative examination of the abdominal cavity [9].

Computed tomography (CT) is the most widely available and best-validated modality for imagining patients with pancreatic adenocarcinoma. It carries a sensitivity for diagnosis of 89-97% [10]. Legmann has reported that CT scan detected 100% of tumors greater than 15 mm in size but only 67% of tumors 15 mm or smaller [4]. Bronstein reported that only 77% of pancreatic tumors 2 cm or smaller were detected [11]. Imaging from our patient initially revealed no evidence of mass, however 8 months later she was found to have a 4.2 cm tumor. This could represent either an unusually rapid presentation of a pancreatic mass or simply exhibit a case of CT imaging being unable to detect a small lesion. In light of the patient's abdominal pain at time of initial presentation, her symptoms may have been attributed to the pancreatic pathology or another biliary etiology (cholelithiasis). At the time of detection, however, the patient was a surgical candidate and underwent a successful pancreaticoduodenectomy. Her operation was performed using the six-step method described by Evans et al [12,13].

The patient involved in this case report had a CT scan performed 8 months prior to the presentation and diagnosis of pancreatic cancer. The initial scan was interpreted as *normal *and during this short interval there was obvious progression to pancreatic adenocarcinoma. This unique clinical scenario is not well described, particularly in cases involving variant anatomy. Gangi et al. reported their institutional experience with abdominal CT scans and its use to detect pancreatic cancer before its clinical diagnosis[14]. In their report, radiologists reviewed CT scans in pancreatic cancer patients that were obtained before histologic diagnosis and CT scans in control subjects. The scans were divided into groups on the basis of the time interval preceding cancer diagnosis (0-2, 2-6, 6-18, or > 18 months). Radiologists agreed that CT findings definite or suspicious for pancreatic cancer were present in 50% of the scans obtained 2-6 and 6-18 months before the diagnosis of pancreatic cancer, but noted such CT findings in only 7% of the scans obtained more than 18 months before diagnosis.

CT can detect a significant proportion of asymptomatic incidental pancreatic tumors before the clinical diagnosis of pancreatic cancer. Pancreatic duct dilatation and cutoff are early findings associated with the development of pancreatic cancer and can be detected on CT with a high degree of reproducibility. These finding as well as mass effect and atrophic parenchyma can be particularly important in detecting pancreatic tumors which can present as isoattenuating on CT [15,16]. Retrospective review of our patient's CT scan did show some pancreatic atrophy which may have been a sign of early pancreatic cancer. However, atrophy alone can be found in numerous benign pancreatic conditions and prospective use of subtle radiographic signs need further refinement.

Improved detection of smaller pancreatic lesions will undoubtedly improve our ability to detect and potentially cure early stage pancreatic neoplasms [17]. There continues to be improvement in detection modalities with the development of contrast-enhanced ultrasound and optical coherence tomography which uses infrared light to produce images showing promise as potential future imaging adjuncts [18].

SI is a rare condition with concomitant pancreatic cancer being even rarer. Despite the rarity, pancreaticoduodenctomy can be safely and successfully performed in these patients who present with rescectable disease provided careful consideration to the anatomy is made [19]. Radiologic detection of early stage pancreatic cancer is paramount to improving survival as surgical resection offers the only chance of long-term cure and special attention needs to be paid in patients with aberrant anatomy. Despite the recent improvements, a reliable means to detect of smaller pancreatic tumors is necessary if early detection of this aggressive malignancy is to translate into improved survival.

## Competing interests

The authors declare that they have no competing interests.

## Authors' contributions

ELS and CJW reviewed the literature and wrote the case report.

## Informed Consent

Written informed consent was obtained from the patient for publication of this case report and accompanying images. A copy of the written consent is available for review by the Editor-in-Chief of this journal.
